# From equilibrium to dynamic metabolic imaging: the evolving role of magnetic resonance spectroscopic imaging in neurodegenerative disorders

**DOI:** 10.1186/s12967-026-09084-z

**Published:** 2026-10-10

**Authors:** Marta M. Pokotylo, Josh P. Peters, Aaron Diercks, Farhad Haj Mohamad, Norbert Brüggemann, Andrey N. Pravdivtsev, Jan-Bernd Hövener, Jannik Prasuhn

**Affiliations:** 1 Department of Neurology, Section of Movement Disorders, University of Luebeck and University Hospital Schleswig-Holstein, Campus Luebeck, Ratzeburger Allee 160, 23538 Luebeck, Germany; 2 Center for Brain, Behavior, and Metabolism, University of Luebeck, Luebeck, Germany; 3 Section of Biomedical Imaging, Molecular Imaging North Competence Center, Department of Radiology and Neuroradiology, University Hospital Schleswig-Holstein, Kiel University, Kiel, Germany; 4 Institute of Neurogenetics, University of Luebeck, Luebeck, Germany; 5 Department of Neurology, Johns Hopkins University School of Medicine, Baltimore, MD USA; 6 F.M. Kirby Research Center for Functional Brain Imaging, Kennedy Krieger Institute, Baltimore, MD USA

**Keywords:** Magnetic resonance spectroscopy (MRS), Magnetic resonance spectroscopic imaging (MRSI), Hyperpolarization, Neurodegenerative diseases, Metabolic modeling

## Abstract

**Background:**

Dysregulated brain metabolism is increasingly recognized to play a significant role in the development and progression of neurodegenerative diseases (NDDs). Conventional imaging methods, such as positron emission tomography (PET), are limited to assessing tracer uptake and offer no insight into its downstream metabolic fate. Additionally, PET provides no insight into dynamic metabolic fluxes and turnover, which reflect biochemical processes. In contrast, magnetic resonance spectroscopy and spectroscopic imaging MRS(I) allow non-invasive, spatially resolved in vivo investigation of specific metabolites, without the use of radioactive tracers.

**Main body:**

^1^H-MRS(I) is one of the most widely utilized techniques due to its accessibility, while heteronuclear approaches, including ^2^H-, ^13^C-, ^15^N-, and ^17^O-MRS(I), broaden MRS(I)’s utility by expanding the scope of accessible metabolites. Technical and methodological advances in nuclear spin hyperpolarization-enhanced MRI further improve signal-to-noise ratio, enabling in vivo detection of low-abundance metabolites. Furthermore, revival of ^2^H-MRS(I) has opened new avenues for dynamic metabolic investigation without hyperpolarization. In this review, we provide a conceptual framework for transitioning from a static to a dynamic MRS(I) approach and discuss key considerations for tracer selection, nuclear spin hyperpolarization methods, acquisition hardware, reconstruction, preprocessing, and metabolic modeling. We outline major methodological and translational challenges, including low signal-to-noise ratio, field strength, and vendor hardware variability, as well as a lack of standardization across acquisition, reconstruction, preprocessing, and modeling strategies.

**Conclusion:**

Dynamic MRS(I) provides a promising foundation for in vivo brain metabolism investigation in NDDs, offering mechanistic insights into metabolic fluxes, reprogramming, and bottlenecks. Although dynamic MRS(I) is limited to a few research institutions, ongoing technical and methodological advances are improving its feasibility, biochemical specificity, and broader implementation. Standardized and harmonized acquisition, reconstruction, and preprocessing protocols are necessary to enable broad clinical implementation of these approaches. Nevertheless, the shift from static to dynamic MRS(I) has great potential to advance biomarker and diagnostic tool development, patient stratification, and longitudinal disease monitoring in NDDs, provided that current challenges and limitations are sufficiently addressed.

## Introduction

Neurodegenerative diseases (NDDs) are a group of disorders characterized by progressive and irreversible neuronal loss, estimated to affect approximately 57 million people worldwide [1]. Despite NDDs’ prevalence, we still lack disease-modifying therapies, while symptomatic treatments yield partially effective and variable responses. The development of therapeutics, diagnostics, and monitoring tools is hampered by the lack of in vivo biomarkers that capture the dynamic processes underlying neurodegeneration and disease progression. Brain metabolism dysregulation is increasingly recognized to be a major contributor to the onset and progression of NDDs such as Alzheimer’s, Parkinson’s, Huntington’s diseases (AD, PD, HD, respectively), and amyotrophic lateral sclerosis (ALS) [2–5].

Despite the importance of brain metabolism, current neuroimaging approaches for its in vivo investigation remain limited. Positron emission tomography (PET) with ^18^F-Fluorodeoxyglucose (^18^FDG) is a conventional approach for investigating glucose utilization, quantifying ^18^FDG uptake as a surrogate marker of metabolic activity. However, PET only detects the accumulation and spatial distribution of ^18^FDG (or another tracer), as it does not differentiate between individual downstream metabolites or resolve metabolic pathways. In turn, magnetic resonance spectroscopy and spectroscopic imaging (MRS(I)) provide spatially and chemically resolved images without radiolabeled tracers. MRS(I) is the only currently available technique that enables non-invasive, in vivo imaging of metabolites, with or without labeled tracers [6]. Unlike PET, tracer-based MRS(I) provides complementary insights into the tracer’s downstream fate, potentially with temporal resolution; however, its broader adoption has been hampered by low signal-to-noise ratio (SNR). MRS(I) targets multiple nuclei (e.g., ^13^C and ^31^P), yet its low gyromagnetic ratio and tissue concentration result in much lower SNR than conventional ^1^H. This can be addressed with isotopically labeled tracers, thereby increasing SNR while reducing background noise. In some cases, signal sensitivity can be enhanced using nuclear spin hyperpolarization-enhanced magnetic resonance imaging (MRI), also known as hyperpolarized metabolic imaging (HMI), thereby increasing signal intensity. Importantly, both dynamic MRS(I) and HMI offer insights into metabolic fluxes and turnover rates, providing complementary perspectives beyond conventional PET and potentially greater sensitivity to subtle, early disease-related alterations.

Dynamic measurements are essential for biological interpretation, allowing assessment of the dynamic process itself rather than its outcome and providing insights into compensatory, maladaptive, and pathological metabolic processes in disease progression. MRS(I)’s capacity to dynamically probe brain metabolism remains underutilized due to low SNR and technical complexity, thereby providing limited insight into the dynamics of metabolic alterations across various NDDs.

This review examines the current status and potential applications of heteronuclear MRS(I) in NDDs research. We discuss both conventional (equilibrium) MRS(I) and HMI approaches, highlighting which aspects of cellular metabolism can be assessed in vivo. We emphasize the need for a practical transition from static to dynamic MRS(I) and outline key technical and biological considerations. We briefly mention the major limitations and challenges associated with signal sensitivity and the biological interpretation of dynamic MRS(I). By integrating the technical and biological principles of these techniques, we aim to present heteronuclear MRS(I) as a promising framework for advancing mechanistic insights into metabolic reprogramming in NDDs and for guiding biomarker development, patient stratification, and longitudinal monitoring of NDDs.

## The utility of brain metabolic assessment in neurodegenerative diseases

**Fig. 1:**
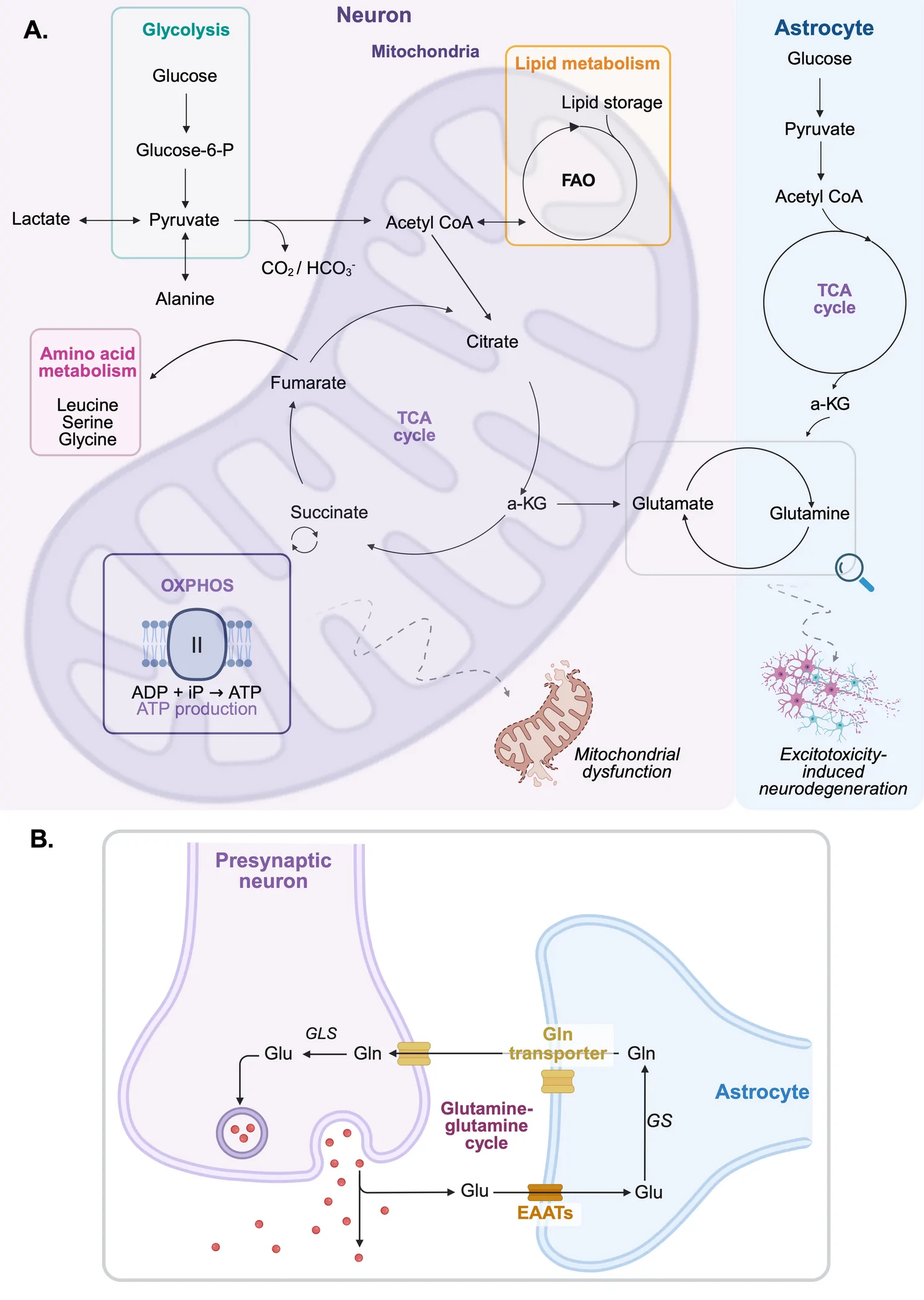
Schematic representation of major metabolic pathways implicated in the pathogenesis of neurodegenerative disease and assessable using MRS(I). (**A**) The figure summarizes key metabolic pathways, including glucose, lipid, and amino acid metabolism within neurons. In neurons, glucose is metabolized through glycolysis to pyruvate, which can be converted to lactate or alanine or enter mitochondria as acetyl-CoA. Acetyl-CoA can be directed towards FAO and lipid storage or enter the TCA cycle, which is coupled to OXPHOS for ATP production. TCA cycle is connected to amino acid metabolism (leucine, serine, glycine). In astrocytes, glucose is metabolized similarly, through glycolysis and the TCA cycle. In both neurons and astrocytes, TCA cycle-derived a-KG can support the glutamate-glutamine cycle. Disruptions in these metabolic pathways may cause downstream mitochondrial dysfunction and excitotoxicity-induced neurodegeneration, as indicated by dashed arrows. (**B**) The panel provides a more detailed explanation of the glutamine-glutamate cycle at the synapse. Glutamate is released from the presynaptic neuron into the synaptic cleft and is taken up by astrocytes through EAATs. Glutamine synthetase converts glutamate to glutamine, which is transported back to the presynaptic neuron via a glutamine transporter. Glutamine is then converted to glutamate by glutaminase for repacking into synaptic vesicles. ADP/ATP: adenosine di/triphosphate. a-KG: alpha-ketoglutarate. CoA: coenzyme A. EAATs: excitatory amino acid transporters. FAO: fatty acid β-oxidation. GLS: glutaminase. Gln: glutamine. Glu: glutamate. GS: glutamine synthetase. iP: inorganic phosphate. MRS(I): magnetic resonance spectroscopy (spectroscopic imaging). OXPHOS: oxidative phosphorylation. TCA: tricarboxylic acid

Metabolism is a complex of interconnected biochemical processes that produce the energy required for neuronal function, with glucose as the brain’s primary energy substrate (Fig. 1) [7]. Glucose is metabolized through glycolysis, the tricarboxylic acid (TCA) cycle, and oxidative phosphorylation (OXPHOS) to generate adenosine triphosphate (ATP), the brain’s primary energy currency. Subsequently, other metabolic processes, including fatty acid β-oxidation (FAO), the glutamate-glutamine cycle, and amino acid synthesis, converge with glucose metabolism, forming a complex interlinked biochemical network. Dysregulated brain metabolism in NDDs manifests as dysfunction of mitochondrial energy production, redox homeostasis, and neuronal-glial metabolic coupling (such as lactate shuttling and glutamate-glutamine cycles), metabolic reprogramming from OXPHOS to glycolysis, and reduced metabolic flexibility, i.e., the ability to switch between metabolic pathways to maintain energy homeostasis [2–5]. As these metabolic processes are vital for maintaining neuronal functioning, they emerge as promising candidates for biomarkers of dynamic processes that play pathological or compensatory roles preceding irreversible neuronal loss and the clinical manifestation. Furthermore, brain metabolism is closely linked to excitotoxicity and neuroinflammation, both of which are widely implicated in NDDs [8–10].

Given the extensive preclinical and clinical evidence of disrupted metabolism in NDDs, the rationale for its research is well justified, yet we lack meaningful, sensitive, and specific approaches for in vivo dynamic probing. From a clinical perspective, brain metabolism is not routinely assessed, primarily due to the lack of effective techniques. Currently, PET is the only non-invasive imaging modality in clinical practice for investigating in vivo brain metabolism, though it has its own limitations, as discussed elsewhere [11]. An additional approach is ex vivo nuclear magnetic resonance (NMR) assessment of patients’ serum, cerebrospinal fluid (CSF), and urine to characterize their chemical composition. Although systematic reviews have demonstrated high diagnostic accuracy of these approaches, their dynamic insights remain limited [12]. Ex vivo NMR-derived metabolite quantification limits our understanding of metabolite uptake, turnover, pathway-specific utilization, equilibration, and kinetics, and does not explain the mechanistic processes underlying the observed final metabolite concentrations. Furthermore, available NMR techniques fall short of distinguishing brain- and peripheral-derived metabolite sources in body fluids.

In turn, MRS enables dynamic quantification of metabolites and their fluxes within a predefined region defined by voxel placement, whereas MRSI goes beyond this approach by acquiring data from multiple voxels, allowing quantification and visualization of their spatial distribution across a collection of voxels, and the creation of metabolic maps. The spatial, temporal, and chemical resolutions are interdependent and often restricted to scales of cubic centimeters (spatial resolution, typically 1–8 cm^3^), minutes (temporal resolution), and millimolar concentrations (chemical sensitivity) [13]. MRS(I) can detect nuclei with nonzero nuclear spin, and the most commonly used nuclei have high natural abundance, such as ^1^H (99.98%) and ^31^P (100%). Nuclei with low natural abundance are also targeted, including ^2^H (0.0156%) and ^13^C (1.1%), using isotopically enriched molecular tracers (e.g., [1-^13^ C]glucose, [^2^H_7_]glucose, [1,2-^13^C_2_]acetate), thereby enabling in vivo mapping of specific metabolic pathways. Given the relatively large number of nuclei that can be detected with MRS(I), it emerges as a versatile tool for specifically observing chemical pathways of interest, thereby increasing its specificity compared to PET. Continuous technological advances, including the development of hybrid magnetic resonance approaches combining MRI acquisition for structural insights along with ^1^H-MRS(I) and deuterium (^2^H)-MRS(I) measurements, as well as improved radiofrequency coils, optimized sequences, and reconstruction techniques, further enhance MRS(I)’s sensitivity, increasing its applicability [14–19].

Under favorable physical conditions, some nuclear spins can be boosted from the parts per million (ppm) range to near unity, a process known as nuclear spin hyperpolarization, enabling second-time temporal resolution with µmol/L sensitivity in vitro and in vivo [20–23]. The probe’s hyperpolarization enables detection of metabolic products that are otherwise invisible at thermal equilibrium (non-hyperpolarized), without the need for extensive averaging across multiple low-SNR scans to enhance the signal [24]. HMI combines the benefits of the conventional MRS(I) approach, including wide chemical shift dispersion and a much lower background signal from endogenous molecules, with greatly enhanced signal sensitivity and SNR, albeit only for a limited duration.

Combining MRS(I) with isotopically labeled tracers (such as ^2^ or ^13^ C) enables assessment of specific metabolic pathways with greater sensitivity and reduced background noise. For example, glucose-based tracers can be used to investigate global brain metabolism, metabolic reserves, and pathway utilization, demonstrating how glucose is processed under different physiological and pathological conditions. Pyruvate-based tracers allow probing glycolytic-mitochondrial coupling, including pyruvate-to-lactate conversion and pyruvate uptake in oxidative metabolism via the TCA cycle, as indicated by glutamate and glutamine labeling. Acetate- and amino acid-based tracers assess glia-weighted and neurotransmitter-related aspects of metabolism, such as the glutamate-glutamine cycle and the role of neuronal-glia coupling in the brain’s metabolism.

## Application of heteronuclear magnetic resonance spectroscopy and spectroscopic imaging to assess metabolism in neurodegenerative diseases

### Proton magnetic resonance spectroscopy and spectroscopic imaging (^1^H-MRS(I))

**Fig. 2:**
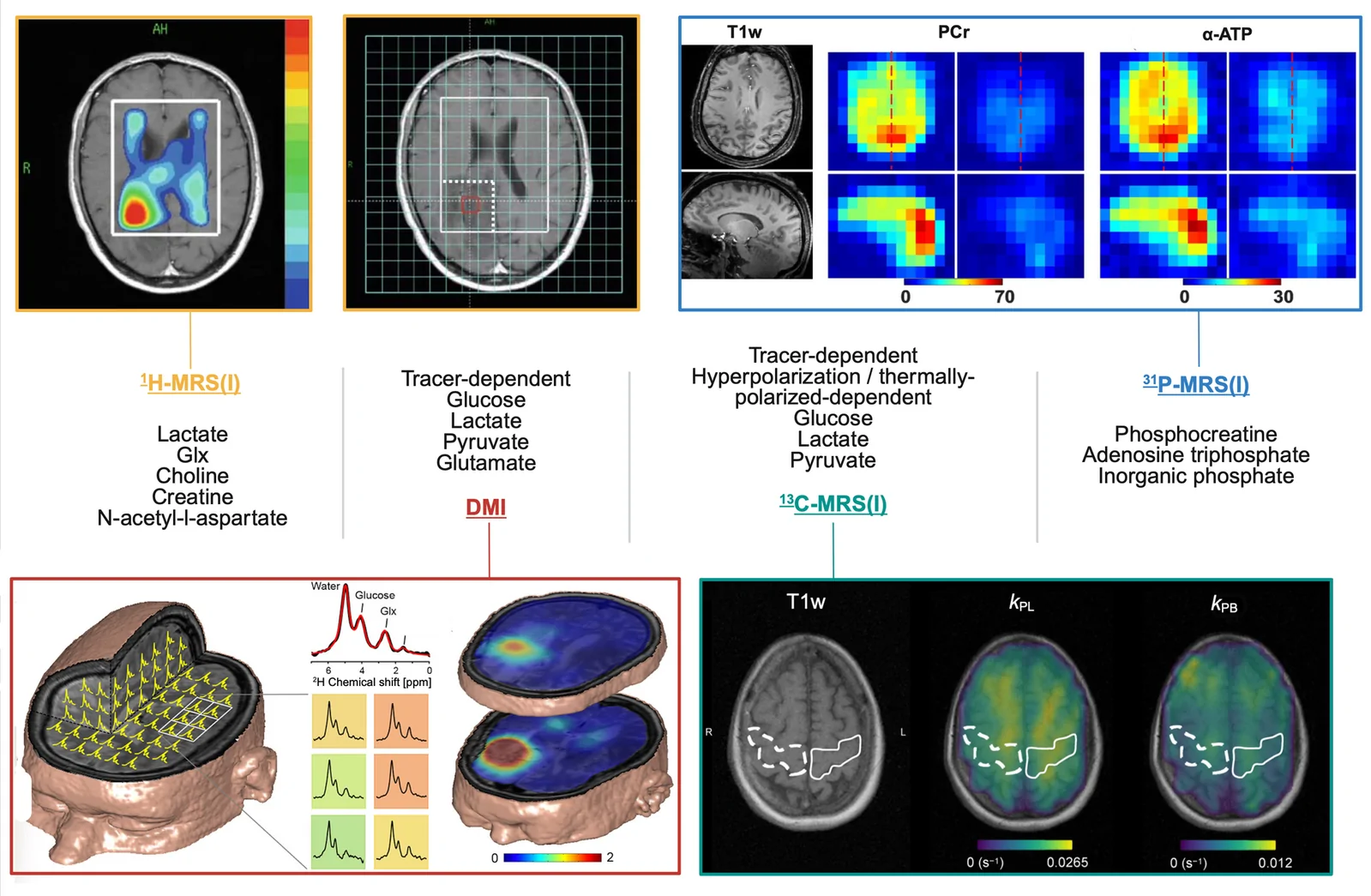
Representative metabolic maps obtained using heteronuclear MRSI. The figure illustrates metabolic maps containing spatial and biochemical information obtained with ^1^H-MRSI (in orange), DMI (in red), hyperpolarized ^13^C-MRSI (in green), and ³¹P-MRSI (in blue). ^1^H-MRSI: a voxel grid overlaid on an anatomical image is presented and used to obtain a regional metabolic map of the Cho/NAA ratio. Adapted from [28]. DMI: 3D representation of a voxel grid overlaid on anatomical images used to obtain regional maps of the lactate/Glx ratio. Adapted from [15]. Hyperpolarized ^13^C-MRSI: Representative T1-weighted anatomical image with corresponding metabolic maps of *k*_PL_ and *k*_PB_. Adapted from [29]. ^31^P-MRSI: Representative T1-weighted anatomical image with corresponding PCr and α-ATP metabolic maps. Adapted from [30]. α-ATP: alpha-adenosine triphosphate. Cho: choline. DMI: deuterium metabolic imaging. Glx: combination of glutamate and glutamine. *k*_PB_: rate constant of pyruvate-to-bicarbonate conversion. *k*_PL_: rate constant of pyruvate-to-lactate conversion. MRSI: magnetic resonance spectroscopic imaging. NAA: N-acetyl-L-aspartate. PCr: phosphocreatine. T1w: T1-weighted

^1^H-MRSI is the most common approach as it has the highest equilibrium sensitivity and minimal hardware requirements [25]. The main disadvantages of ^1^H-MRS(I) are the high background signal from the highly abundant water and macromolecules and the narrow chemical shift range (0–10 ppm), which complicate quantitative analysis and limit resolution. Nevertheless, ^1^H-MRS(I) remains the most widely used technique for non-invasive, in vivo imaging of metabolites without ionizing radiation. Examples include N-acetyl-L-aspartate (NAA), creatine, myo-inositol, glutamate, glutamine, lactate, and other metabolites (Table 1; Fig. 2) [26, 27]. While absolute quantification of metabolite concentrations is a major topic of interest, relative quantification based on metabolic ratios (e.g., NAA-to-creatine and lactate-to-creatine ratios) is routinely used to define normative values in single-voxel MRS and multi-voxel MRSI [26]. ^1^H-NMR has been instrumental in uncovering the relationship between disrupted metabolism and disease pathology across a spectrum of chemically and genetically induced animal models of PD, HD, and spinocerebellar ataxia type 3 [31–40]. Utilizing ^1^H-MRS(I), dysregulation in glutathione and glutamate/glutamine balance was identified in patients with AD, PD, ALS, and even progressive supranuclear palsy [41–47]. Frequently, ^1^H-MRS(I) has been used to investigate the relationship between brain metabolism and cognitive state in AD and PD, with observed correlations between NAA, total choline, and myoinositol and cognitive state [48–53]. As ^1^H-MRS(I) has been available for several decades, the volume of research utilizing this technique is vast, with numerous reviews of its use in NDDs research published [54, 55]. Nevertheless, ^1^H-MRS(I) has been predominantly used in cross-sectional study designs, with only one study longitudinally examining metabolic alterations and their association with cognitive state in patients with ALS [56]. The longitudinal assessment revealed positive correlations between NAA/total creatine and cognitive performance in patients with ALS, with metabolite levels remaining stable over time (Table 2) [56]. This study demonstrates that a mechanism distinct from absolute metabolite concentrations drives the observed phenotypes, warranting further investigation.

**Table 1:** Properties, applications, and limitations of nuclei used in magnetic resonance spectroscopy and spectroscopic imaging

Target Nucleus	Relaxation times in vivo	Gyromagnetic ratio (MHz/T)	Target metabolites & applications	Advantages	Major limitations
^1^H	T_1_ ≈ 1–3 s T_2_ ≈ 50–120 ms	42.58	NAA Choline Creatine Lactate Myo-inositol Glu Gln	Widely available on clinical scanners; No labelled tracer required	Water and lipid contamination; Narrow chemical shift
^2^H	T_1_ ≈ 0.1–0.3 s T_2_ ≈ 1–50 ms	6.54	Glucose uptake Lactate production TCA cycle activity Glu-Gln metabolism (Glx) Oxidative vs. anaerobic metabolism	Short T1 = faster acquisition; Low natural background signal = higher specificity	Tracer needed; Long acquisition times; Low spatial resolution; Limited sensitivity
^13^C	T_1_ ≈ 5–20 s T_2_ ≈ 20–200 ms	10.71		Wide chemical shifts = superior spectral resolution; Low natural background signal = pathway-specific tracing	Tracer needed; Low intrinsic sensitivity; Long T1 = long acquisition times; High technical complexity
^15^N	T_1_ ≈ few s – over 500 s T_2_ ≈ 50 ms – several s	-4.32	Redox state Amino acid metabolism Nicotinamide metabolism	Wide chemical shifts = superior spectral resolution	Tracer needed; Extremely low intrinsic sensitivity; Long acquisition times
^17^O	T_1_ ≈ 1–10 ms T_2_ ≈ 1–5 ms	-5.77	Cerebral oxygen uptake OXPHOS Mitochondrial respiration	Fast T1 = rapid dynamic sampling	Tracer needed; Low sensitivity; High technical complexity; Limited clinical availability; Safety regulations
^31^P	T_1_ ≈ 2–6 s T_2_ ≈ 30–100 ms	17.24	Phosphocreatine Inorganic phosphate Adenosine triphosphate Bioenergetics metabolism	100% natural abundance; Relatively high sensitivity among heteronuclei	Low spatial resolution; Long acquisition times

Gln: glutamine. Glu: glutamate. Glx: combination of glutamate and glutamine resonance signals. NAA: N-Acetyl-L-Aspartate. OXPHOS: oxidative phosphorylation. TCA: tricarboxylic acid

**Table 2:** Representative applications of magnetic resonance spectroscopy and spectroscopic imaging in patients with neurodegenerative disease

Disease	Modality	Tracer used	Field strength	Main metabolic finding
Alzheimer’s disease	^1^H-MRS	N/A	1.5 T	Decreased Glu and NAA/Cr in the posterior cingulate and hippocampus in Alzheimer’s disease. Glu correlated with cognitive decline. [57].
Parkinson’s disease	^1^H-MRS	N/A	3 T	Significantly reduced GABA levels in the midbrain and glutathione in left striatum [43].
Parkinson’s disease	^1^H-MRS	N/A	3 T	Reduced NAA/Cho and NAA/Cr rations in the substantia nigra and basal ganglia, which correlated with UPDRS score [58].
Amyotrophic lateral sclerosis	^1^H-MRS	N/A	3 T	Positive correlation between NAA/total creatine and cognitive performance [56].
Progressive supranuclear palsy	^1^H-MRSI	N/A	3 T	Significantly reduced global NAA in patients with supranuclear palsy compared to idiopathic Parkinson’s disease [47].
Healthy volunteers	^1^H-MRSI	[6,6-^2^H_2_]glucose	7 T	Negative correlations between Glc and time in gray and white matter, with faster metabolic activity in gray matter [59].
Alzheimer’s disease	^2^H-MRS	[6,6-^2^H_2_]glucose	3 T	Reduced medial temporal lactate and Glx in patients with Alzheimer’s disease [60].
Alzheimer’s disease	^2^H-MRSI	[6,6-^2^H_2_]glucose	3 T	Reduced Glx/Glc in patients with Alzheimer’s indicating impaired oxidative metabolism [61].
Alzheimer’s disease	^13^C-MRS	[1-^13^ C]glucose	1.5 T	Delayed ^13^C enrichment of Glu2 indicating reduced glucose oxidation & reduced Glu2/Glu4 enrichment ratios indicating impaired neuronal metabolism in patients with Alzheimer’s disease [62].
Amyotrophic lateral sclerosis	HP ^13^C-MRSI	[1-^13^ C]pyruvate	3 T	40% increase in pyruvate-to-lactate conversion rate, which correlated with patient’s symptoms [29].
Healthy volunteers	^17^O-MRSI	^17^O gas	3 T	Observed higher cerebral metabolic rate of oxygen uptake occipital gray matter compared to frontal and parietal regions [63].
Parkinson’s disease	^31^P-MRS	N/A	3 T	Levodopa/benserazide treatment resulted in reduction of high-energy phosphorus-containing metabolites in the basal ganglia of patients with Parkinson’s disease [64].
*COQ8a*-related ataxia	^31^P-MRS	N/A	3 T	Higher energy metabolites levels in patients post Q10 supplementation, indicating that bioenergetic state may predict treatment response [65].

The table summarizes representative studies that used X-nuclei magnetic resonance spectroscopy and spectroscopic imaging in patients with neurodegenerative diseases published in the last 5 years. Given the extensive literature available, the list of ^1^H- and ^31^P-MRSI is not full. Cho: choline. Cr: creatine. Glc: glucose. Glu, Glu2/Glu4: glutamate. Glx: a combination of glutamate and glutamine. HP: hyperpolarized. MRS: magnetic resonance spectroscopy. MRSI: magnetic resonance spectroscopic imaging. NAA: N-acetyl-L-aspartate. UPDRS: Unified Parkinson’s disease Rating Scale

Since all MRI systems include ^1^H observation capabilities, ^1^H-MRS(I) requires no additional hardware, making it the most cost-effective technique; yet its utilization for dynamic investigations remains limited. Dynamic and time-resolved ^1^H-MRS(I) is feasible with echo-planar imaging (EPI), functional MRS(I) paradigms, and compressed sensing [66, 67]. Additionally, the needed software for reproducible spectral quantification and kinetic modeling of dynamic time-resolved measurements is already in place, as exemplified by the open-source Osprey [68]. However, the main limitations of the spectral overlap, low sensitivity to subtle metabolic shifts, and lack of standardized acquisition and processing workflows remain.

### ^2^H (deuterium) metabolic imaging (DMI)

The low natural abundance and the use of isotopically enriched tracers help address signal contamination in ^1^H-MRS(I). ^2^H has been of interest since the early days of magnetic resonance and relies on exogenous administration of ^2^H-labeled tracers [69–71]. The sensitivity of deuterium metabolic imaging (DMI) scales supralinearly with magnetic field strength; thus, imaging quality depends on available hardware [72]. Although DMI requires ^2^H-related hardware, deuterium labeling can be indirectly detected using conventional ^1^H-MRS(I), as exchange between deuterium-labeled and unlabeled molecules produces position-specific signal decreases at the corresponding ^1^H resonance. These indirect readouts detected dynamic, exchange-driven signal changes for glutamate and glutamine, which remain difficult to differentiate. Additionally, ^2^H-label-induced changes in the GABA spectral region have also been assessed, but with no statistical significance, potentially reflecting higher measurement variance due to non-edited ^1^H-MRSI [73]. This approach has been utilized in healthy volunteers at 3 Tesla (T), demonstrating its feasibility and the dynamic detection of tracer-induced metabolic changes as small as 10–20% [59].

The ongoing discovery and validation of new ^2^H-labeled tracers for targeting various chemical pathways continue to expand DMI’s utility. Examples include the feasibility of simultaneously using [^2^H_7_]glucose and [^2^H_2_]fructose to assess tissue energy substrate preferences [74, 75], which is relevant to NDDs and supported by extensive evidence of consistent cerebral glucose hypometabolism. Similarly, [3,4,4,4]-^2^H_4_-β-hydroxybutyrate [76] can be used to assess the compensatory effects in ketone body utilization in response to glucose hypometabolism, while glucose analogs such as [3-O-C^2^H_3_]glucose [77] can be used to investigate glucose uptake, with the ultimate goal of understanding the mechanistic processes underlying the observed disrupted glucose metabolism in NDDs [2–5]. Furthermore, [^2^H_3_]acetate is being validated as a tracer preferentially utilized by microglia [15, 69, 78], thereby enabling in vivo cell-specific metabolic differentiation, which is highly relevant to inflammatory conditions observed across all NDDs [10].

DMI’s feasibility has been demonstrated with whole-brain coverage in healthy volunteers across a spectrum of field strengths, including 3 T, 7 T, and 9.4 T [61, 79, 80]. The high reproducibility of DMI findings in healthy volunteers at 3 T further validates DMI’s robustness [81]. DMI has been used in patients with AD, revealing decreased glucose metabolism in the medial temporal lobe and providing complementary insights to those from simultaneously acquired PET [60]. Additional studies in patients with AD identified impaired oxidative glucose metabolism at 3 T; although reduced glucose metabolism was reported, the underlying mechanistic alterations were not characterized, underscoring the potential value of dynamic metabolic measurements [61].

Technical improvements, such as density-weighted concentric ring trajectory (CRT) sampling combined with DMI at 7 T, further advance high-resolution metabolic mapping with accurate differentiation between gray and white matter [82]. Notably, a recently published study demonstrated the ability to perform dynamic DMI measurements at 7 T with whole-brain coverage and to subsequently model and assess glucose uptake, lactate production, and TCA cycle kinetic rates using a single tracer, deuterated glucose [83]. Furthermore, DMI has been used in a multimodal setting, combined with ^13^C-HMI, which showed that although both techniques probe similar metabolic pathways, their combination provides complementary insights in healthy volunteers at 3 T [84].

Several technical limitations of DMI remain to be addressed, including low SNR, the need for high tracer doses, long acquisition times, and broad, overlapping spectral peaks. The limited availability of ^2^H hardware and the need for extensive expertise in its operation remain among the biggest barriers to its widespread clinical translation, as most clinical MRI systems currently lack DMI-compatible transmit/receive options and require higher field strengths for robust spectral acquisition.

### ^13^Carbon magnetic resonance spectroscopy and spectroscopic imaging (^13^C-MRS(I))

Carbon (^12^C) is highly abundant in all biological systems, whereas the magnetically active ^13^C isotope has a natural abundance of only ~ 1.1%, which limits the intrinsic SNR for low-concentration brain metabolites. This limitation can be overcome by administering isotopically labeled ¹³C tracers, enabling label incorporation into downstream glycolytic and TCA intermediates and improving their detectability [85–87]. ^13^C offers a wider chemical-shift range (> 200 ppm), i.e., greater metabolite separation, minimal background contamination, and a clearer distinction between glutamate/glutamine than ^1^H-MRS(I) [88]. ^13^C-MRS(I) has a broad application portfolio, as it allows probing various branches of glucose metabolism, including glycolysis, the TCA cycle, and the glutamate-glutamine cycle [89, 90]. The utility of ^13^C-MRS(I) in NDDs research was demonstrated in the early 2000s, revealing reduced glutamate neurotransmission in patients with AD compared with age-matched healthy controls at 1.5 T [62]. However, the evident lack of recent studies using this technique stems from the need for specialized ^13^C equipment, tracers, long acquisition times, low SNR, and technical expertise. Nonetheless, the development of ^1^H-MRS(I)-based acquisition protocols to detect ^13^C-label-induced changes in ^1^H spectra allows its continuous use, mitigating some of the technique’s cost [91, 92].

Despite the use of ^13^C-labeled tracers, the signal may remain low due to low thermal polarization, limiting detection to high-abundance metabolites. However, the spin polarization of the tracers used can be increased using hyperpolarization methods, such as dynamic nuclear polarization (DNP), which is the most widely used for clinical ^13^C-labeled probes [20]. Although the use of parahydrogen-induced polarization (PHIP) for ^13^C-containing probes is less common in biological tissue, its feasibility has been demonstrated in preclinical models, with the hope of clinical translation, thereby reducing costs [93, 94]. ^13^C-HMI has already been applied in vivo, mainly in cancer and traumatic brain injury studies, where disrupted metabolic function is well established [95–97], with rare use in NDDs research. For instance, hyperpolarized [1-^13^C]pyruvate has been used in mice and minipigs to assess the metabolic consequences of an AD-associated *SORL1* mutation, allowing the detection of downstream lactate and bicarbonate [98]. As *SORL1* encodes Sortilin-related receptor 1, which is involved in mitochondrial trafficking, its mutation disrupts mitochondrial function and has been associated with reduced conversion of [1-^13^ C]pyruvate to downstream metabolites, consistent with dysregulated metabolism in AD [98]. Furthermore, a proof-of-concept study in patients with ALS reported disrupted glucose metabolism, specifically an increased pyruvate-to-lactate conversion rate in the left motor area, which corresponded to increased motor symptom burden, suggesting that increased anaerobic activity may underlie the observed phenotype [29].

^13^C-HMI has been established in various preclinical cancer models and is currently in the initial stages of clinical adaptation; although it remains very restricted to specialized centers due to the high associated costs and technical demands [99, 100]. Despite the slow adoption of ^13^C-HMI across neurological conditions, its high biological relevance [2–5], evidence of multicenter reproducibility, and consensus further support its use in NDDs [101, 102]. Meanwhile, thermally stable ^13^C-MRS(I) offers the advantage of assessing chemical activity over a longer period but is hindered by low intrinsic sensitivity, leading to long acquisition times and low SNR, and is currently limited in clinical utilization.

### ^31^Phosphorus magnetic resonance spectroscopy and spectroscopic imaging (^31^P-MRS(I))

^31^P-MRS(I) takes advantage of ^31^P’s 100% natural abundance, making it one of the most sensitive nuclei for magnetic resonance detection and the second most widely used technique. ^31^P-MRS(I) probes the metabolism of high-energy phosphorus-containing compounds, including ATP, inorganic phosphate (iP), and phosphocreatine (PCr), providing indirect insights into mitochondrial function and cellular respiration. Quantification of ATP reflects the immediately available energy for cellular processes, whereas PCr indicates reserve levels. PCr facilitates ATP generation, releasing Cr and iP in the creatine kinase reaction, which serves as an immediate energy buffer by maintaining relatively stable ATP levels under fluctuating energy demand. As a result of ATP hydrolysis, iP is produced, serving as a marker of elevated energy consumption or impaired OXPHOS, both of which have been shown to be disrupted in NDDs. Recently, ^31^P-MRS(I) has been used to assess mitochondrial dysfunction and investigate the effects of sex and menopause on phosphorus-containing metabolites in AD and PD, demonstrating sex-specific metabolic differences relevant to disease management [103–109]. ^31^P-MRS(I) has been used to assess the efficacy of various therapies, probing for positive and negative metabolic changes, including Vitamin K2, NAD^+^ (nicotinamide adenine dinucleotide, oxidized form) supplementation, ursodeoxycholic acid, MAO-B (monoamine oxidize B) inhibitors, and terazosin in PD, and Q_10_ supplementation in coenzyme Q8A (COQ8A)-related ataxia [109–114]. Notably, ^31^P-MRS(I) assessment of PD symptomatic treatment with L-DOPA revealed differential effects on phosphorus-containing metabolism in the basal ganglia, which have been proposed as a potential mechanism underlying L-DOPA-associated bradykinesia [115].

Given its favorable physical properties, ^31^P requires no specific tracers, yet available literature has mainly focused on single-point measurements, underutilizing its potential for dynamic assessment of phosphorus-containing metabolism. Dynamic ^31^P-MRS(I) measurements have been widely used in skeletal muscle studies [116] and can be applied to probe the brain in NDDs. Yet its utility is limited by the small number of available preclinical studies needed to standardize acquisition and processing.

## Brief note on emerging techniques: ^15^N and ^17^O

### ^15^Nitrogen magnetic resonance spectroscopy and spectroscopic imaging (^15^N-MRS(I))

Similar to ^12^C, naturally abundant ^14^N is magnetic resonance-inactive, whereas its stable ^15^N isotope has favorable physical properties for magnetic resonance detection. The low natural abundance and sensitivity of ^15^N are typically addressed with ^15^N-labeled tracers, which minimize background signal and improve detection specificity. Nevertheless, the ^15^N signal remains inherently weak, which explains the limited number of ^15^N-NMR and MRS(I) studies in NDDs. Despite its limitations, ^15^N-MRS(I) has significant utility for assessing the glutamate-glutamine cycle and the redox state, as reflected in the turnover of the oxidized and reduced forms of nicotinamide adenine dinucleotide (NAD^+^/NADH), a critical regulator of mitochondrial health and energy production [117].

The feasibility of ^15^N-NMR was established in the 1990s in live rat models using various approaches for signal enhancement, including heteronuclear multiple quantum coherence (HMQC) methods, which allowed selective probing of glutamine amide protons and glutamate pools, thereby assessing glutamine synthase activity and glutamine turnover [118]. Further research employed the 3D image-selective in vivo spectroscopy (ISIS) technique, combining nuclear Overhauser enhancement (NOE) and protocol decoupling in [^15^N] leucine-infused rats, allowing detection of [^15^N]ammonium acetate and glutamine pools and establishing the methodology for probing nitrogen metabolism in vivo [119]. Low signal sensitivity can be partially mitigated by hyperpolarization, as ^15^N’s inherently long T_1_ produces a long-lasting hyperpolarized state, suitable for prolonged in vivo measurements. As proof of concept, long-lasting signal detection following the injection of hyperpolarized [^15^N]choline chloride was demonstrated in live rats [120]. Choline is a precursor to the neurotransmitter acetylcholine, whose synthesis is significantly impaired in AD, providing a specific probe for assessing acetylcholine metabolism [121].

Despite technical advances, ^15^N-MRS(I) remains in an early experimental stage and has yet to be used in the context of NDDs. Its widespread use is limited by its inherently low intrinsic sensitivity, low spatial resolution, high experimental cost, long acquisition times, and the lack of validated HMI probes. Additionally, the available evidence is limited to preclinical research, demonstrating feasibility only and with limited human validation, warranting further research.

### ^17^Oxygen magnetic resonance spectroscopy and spectroscopic imaging (^17^O-MRS(I))

MRS(I) also targets oxygen isotopes, with only ^17^O being magnetic resonance-active. ^17^O is introduced via exogenous administration of ^17^O-enriched oxygen, enabling direct assessment and quantification of cerebral oxygen uptake and its conversion to CO_2_ as a marker of mitochondrial respiration. Its use in the R6/2 HD model revealed regional reductions in striatal mitochondrial maximal capacity, linking oxygen metabolism to disease-specific neurodegenerative mechanisms [122]. This study highlights the potential of dynamic, real-time probing of mitochondrial dysfunction in vivo, despite technical limitations. However, its findings should be used with caution, as the ^17^O-NMR measurements were performed in anesthetized animals, which differ in glucose metabolism from non-anesthetized animals, and the impact on oxygen metabolism remains unknown [123–125]. Replicating such findings in healthy volunteers/patients would provide more accurate mechanisms insights and strengthen the technique’s utility and interpretability. ^17^O-MRS(I) has been performed in healthy volunteers at 3 T, successfully quantifying the oxygen uptake rate across multiple regions of interest and providing a framework for its future use [63].

Despite evidence of in vivo feasibility in healthy volunteers and promising preclinical findings, clinical implementation of ^17^O-MRS(I) is hindered by the need for specialized hardware and safety considerations related to handling ^17^O-enriched oxygen. Furthermore, the small number of available publications restricts protocol standardization, leaving ^17^O-MRS(I) in its very early experimental stages.

## From static to dynamic magnetic resonance spectroscopy and spectroscopic imaging

**Fig. 3:**
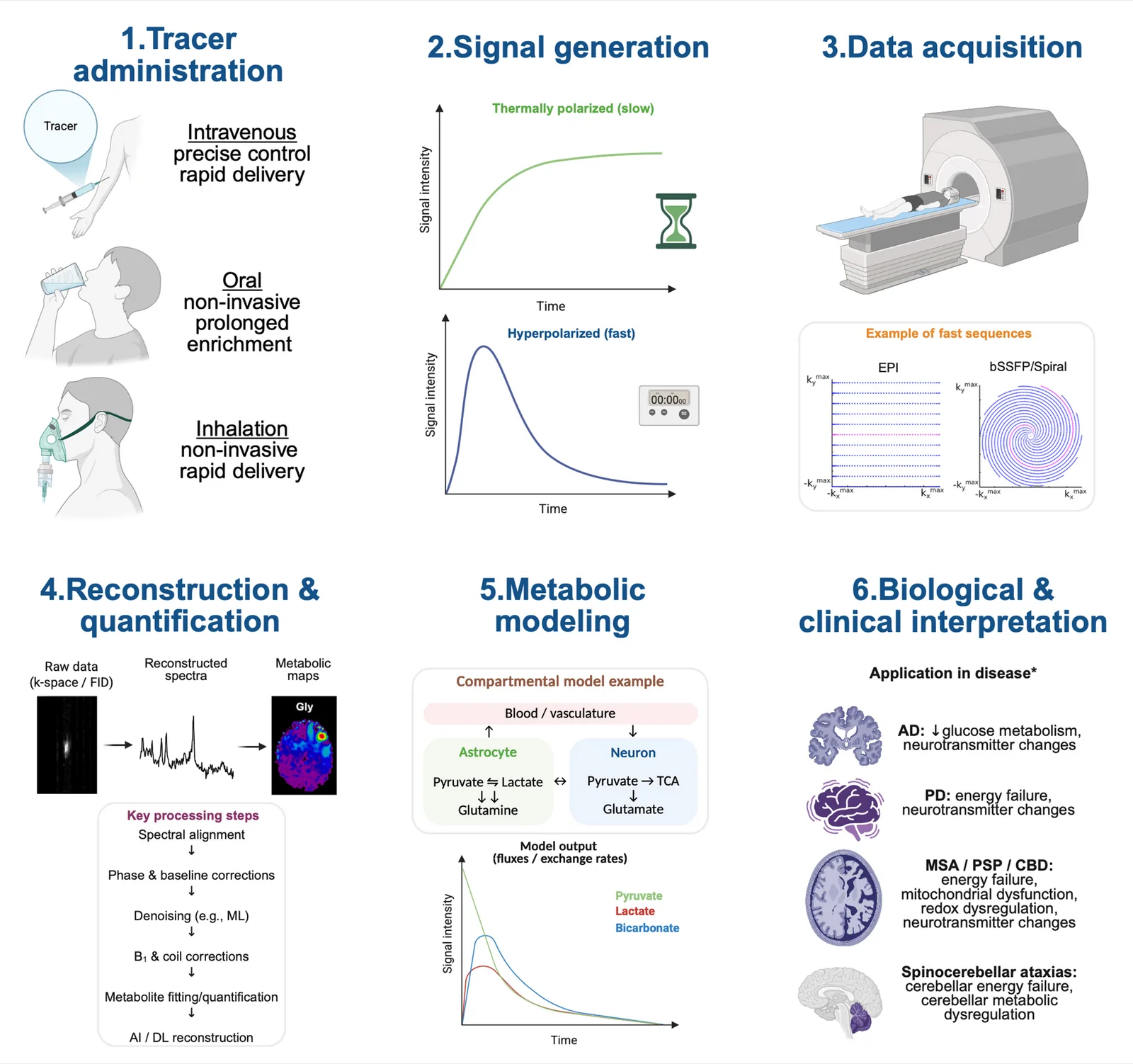
Schematic overview of workflow and key challenges in MRS(I). The figure summarizes the major steps in dynamic magnetic resonance spectroscopy measurements, including: (1) tracer administration, (2) signal generation, (3) data acquisition, (4) reconstruction and quantification, (5) metabolic modeling, and (6) biological and clinical interpretation. We derived images from EPI and bSSFP sequences, raw data, and metabolic maps from Bogner et al. [66]. AD: Alzheimer’s disease. AI: artificial intelligence. bSSFP: balanced steady-state free precession. CBD: corticobasal degeneration. DL: deep learning. EPI: Echo-Planar imaging. FID: free induction decay. ML: machine learning. MRS(I): magnetic resonance spectroscopy (spectroscopic imaging). MSA: multiple system atrophy. PD: Parkinson’s disease. PSP: progressive supranuclear palsy. TCA: tricarboxylic acid cycle

The shift from a static to a dynamic MRS(I) enables the acquisition of time-resolved changes in metabolite signals, either in endogenous metabolites or in an administered isotopically labeled tracer or probe. Dynamic MRS(I) involves repeatedly acquiring spectra of interest over a defined period to quantify metabolic fluxes in vivo. Dynamic MRS(I) measurements are not limited to tracer-based studies and can be used for conventional time-resolved measurements of endogenous metabolites using ^1^H- and ^31^P-MRS(I) acquisitions. The workflow of dynamic MRS(I) is highly dependent on the underlying biological question. For example, whether rapid or slow metabolic processes are investigated, whether the goal is to quantify metabolic fluxes or neurotransmitter cycling, or simply to characterize disease-specific metabolic phenotypes, and whether thermally stable or hyperpolarized probes are necessary. As a result, the experimental workflow is adapted to balance temporal, spectral, and spatial resolutions, accounting for SNR limitations, tracer delivery kinetics, hardware availability, field strength, coil configuration, sequence-specific constraints, and patient population (Fig. 3). Importantly, the biological interpretation of dynamic MRS(I)-derived metabolic fluxes depends on metabolic modeling approaches, which introduce uncertainty arising from model assumptions. Therefore, estimated metabolic fluxes should be interpreted with caution and, where possible, validated with complementary biochemical investigations or ex vivo approaches. In the following section, we provide a brief overview of major technical considerations involved in designing and interpreting dynamic MRS(I) measurements.

### Practical considerations for isotopically labeled tracers and hyperpolarized probes

In tracer-based studies, tracer properties and polarization state affect the temporal quality of dynamic measurements. Following administration of thermally polarized tracers such as [6,6-^2^H_2_]glucose, a prolonged acquisition time (tens of minutes to hours) is needed to observe tracer uptake and metabolic fate, while hyperpolarized probes, such as [3,3,3-^2^H_3_]pyruvate and [1-^13^ C]pyruvate, need rapid acquisition (seconds to minutes) due to the above-mentioned short-lasting polarization [84, 126]. Therefore, HMI is more suitable for investigating rapid metabolic reactions, such as pyruvate-to-lactate conversion, whereas thermally polarized MRS(I) assesses metabolic processes requiring prolonged tracer incorporation and metabolism, such as glucose uptake, glycolysis, and the TCA cycle.

**Fig. 4:**
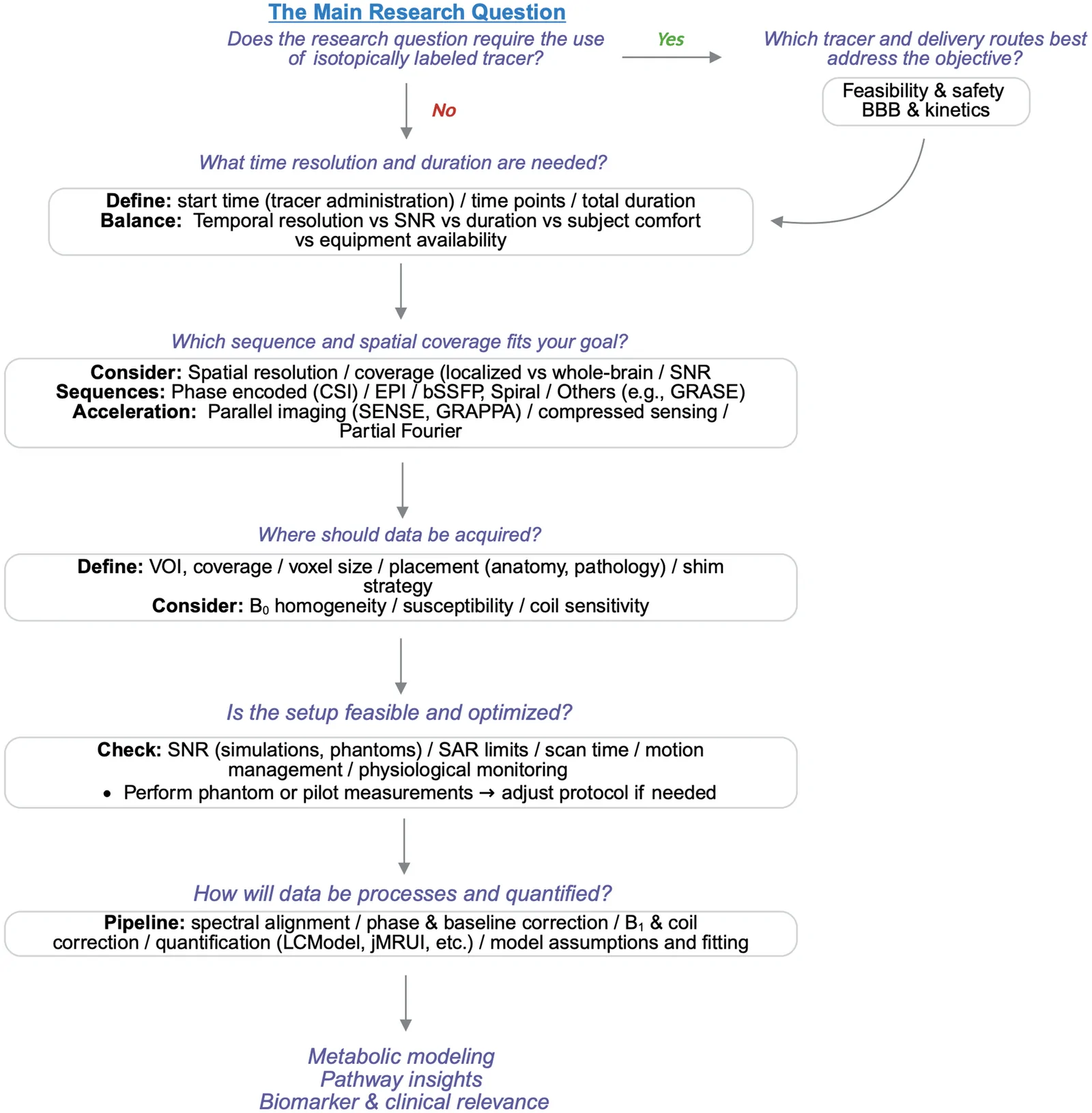
Conceptual framework for designing dynamic hyperpolarized or thermally polarized MRS(I) studies. The figure presents a stepwise workflow for designing and implementing dynamic spectroscopy-based metabolic imaging, outlining factors to consider at each step and overarching considerations. BBB: blood-brain barrier. bSSFP: balanced steady-state free precession. CSI: chemical shift induction. EPI: Echo-Planar Imaging. GRAPA: generalized autocalibrating partially parallel acquisitions. GRASE: gradient and spin echo. MRS(I): magnetic resonance spectroscopy (spectroscopy imaging). SENSE: sensitivity encoding. SAR: specific absorption rate. SNR: signal-to-noise ratio. VOI: volume of interest

The accuracy of dynamic MRS(I)-derived biological interpretation depends on the tracer kinetics incorporated into the metabolic model, which are influenced by the route of administration and its limitations. Intravenous administration allows precise control over tracer dose and infusion rate and is often used in combination with hyperpolarized probes, where rapid delivery is needed, but its invasive nature limits its use in some patient groups, such as elderly participants and patients with NDDs. Conversely, oral administration is less invasive and enables steady, sustained isotopic enrichment, which is common in dynamic studies. Nevertheless, variable absorption, poor temporal resolution, and delayed systemic equilibrium increase interindividual variability and complicate the metabolic modeling. Comparative research demonstrated that oral administration achieves isotopic enrichment levels comparable to those of the intravenous route; however, with greater variability due to intestinal absorption [127, 128]. Additional physiological factors for consideration include the probe’s blood-brain barrier (BBB) permeability, age, sex, cerebral perfusion, cellular transport, and uptake; however, the available literature on these aspects remains limited (Fig. 4) [129, 130]. Furthermore, the MRS(I)-measured voxel contains a highly heterogeneous mixture of neurons, astrocytes, and microglia, complicating compartment-specific assignment of tracer dynamics and fluxes (Fig. 1). Cell-specific differentiation is one of the main limitations of MRS(I), as we are unable to distinguish between observed metabolic processes, such as neuronal versus glial metabolism. Advancements in this direction may be particularly relevant in NDDs with a known differential pattern of neuronal and glial pathology, such as PD and multiple system atrophy (MSA), potentially advancing mechanistic interpretations and disease stratification [131]. Inhalation-based administration is used for tracers such as ^17^O-enriched oxygen, enabling rapid tracer distribution and non-invasive brain uptake. However, this approach is limited by a lack of standardized protocols and safety considerations, and, primarily, by the need for specialized gas-handling infrastructure.

Emerging technical approaches address some of the tracer-related limitations, such as focused ultrasound-based BBB disruption, increasing its permeability, as demonstrated with hyperpolarized [1-^13^ C]pyruvate, allowing faster delivery with minimal side effects in healthy volunteers [132]. As with other therapeutic agents, injected, orally administered, or inhaled tracers and probes must comply with Good Manufacturing Practice (GMP) guidelines set by the relevant authority (e.g., Food and Drug Administration, FDA, or European Medicines Agency) to ensure standardized synthesis and tracer formulation, tracer purity, and endotoxin- and pathogen-free nature [133, 134].

### Production of hyperpolarization: methods, hardware, and limitations

If the research question relies on hyperpolarized probes, additional factors should be considered, including probe production, available equipment, and technical complexity. The production of hyperpolarized probes involves increasing nuclear spin polarization above thermal equilibrium to enhance signal sensitivity for detecting otherwise undetectable biochemical processes [135]. Standard methods for hyperpolarization include DNP [20], PHIP (26), and signal amplification by reversible exchange (SABRE) [136]. DNP is currently the only clinically established technique for HMI in humans [137], while PHIP and SABRE remain at the experimental and preclinical level despite their cost-effectiveness.

In dissolution DNP (dDNP), high electron spin polarization is transferred from stable radicals to target nuclei via microwave irradiation in a glass matrix at temperatures near absolute zero (~ 1.2 K) [138, 139]. The process requires several minutes to hours at cryogenic temperatures, using microwave and radiofrequency radiation, followed by rapid dissolution in a biocompatible solvent, typically buffered saline solution, to create a suitable in vivo hyperpolarized probe [20]. dDNP is an established clinical approach for [1-^13^ C]pyruvate hyperpolarization; however, slow polarization buildup, the need for expensive equipment (~$1–2 million USD), radical removal, rapid probe handling, and sterility control increase the technique’s technical complexity and limit its use to a few institutions.

Hydrogenative PHIP uses the singlet nuclear spin isomer of dihydrogen, called parahydrogen (pH_2_), as a source of spin order [140, 141]. pH_2_ is chemically added to an unsaturated bond in a precursor molecule, thereby transferring its spin alignment to various forms [142]. The most biologically relevant case is when the spin alignment of pH_2_ is transferred to the target ^13^C nucleus, e.g., pyruvate or fumarate [93, 143, 144]. PHIP offers a faster, lower-cost alternative to DNP, as it does not require superconducting magnets [94, 145]. Similar to dDNP, the lifetime of the PHIP-created hyperpolarized state ranges from several seconds to a few minutes and strongly depends on molecular structure and on physical and chemical conditions [146–148]. Although experimental pH_2_-based methods are cost-effective compared to DNP, current *in vivo-*compatible commercial PHIP systems are comparable in cost to commercial dDNP systems. Moreover, PHIP requires substrates containing unsaturated bonds and rapid chemical reactions, resulting in low biocompatibility of currently used catalysts, which must be removed before probe administration, hampering clinical translation. Alternatively, acetone-water mixtures and fluorinated solvents combined with methanol are being investigated, thereby advancing these techniques toward in vivo compatibility [149–151]. Similar to PHIP, SABRE-based approaches leverage pH_2_-derived spin order to transfer polarization to the substrate without permanent chemical modification [152]. As SABRE is widely used in experimental settings and is limited to biologically incompatible probes, it will not be discussed in further detail.

HMI is inherently constrained by rapid signal decay, requiring precise coordination among probe hyperpolarization, administration, and the acquisition window. Despite the short acquisition period, hyperpolarized signal dynamics reflect a complex interplay among tracer delivery, cerebral perfusion, transport kinetics, compartmental redistribution, and enzymatic exchange, rather than an isolated chemical reaction. Therefore, the accuracy of biological interpretation is primarily influenced by modeling assumptions, SNR, polarization variability, and spectral quantification accuracy. Furthermore, the differences in achievable polarization levels and hardware complicate quantitative reproducibility across subjects and centers. These limitations highlight the technical and infrastructure demands. At present, ^13^C-HMI is the most clinically advanced technique, yet it requires further validation, standardization, and multicenter reproducibility assessment before broader implementation.

### Hardware and data acquisition requirements

For all heteronuclear metabolic imaging techniques, whether at thermal equilibrium or in a hyperpolarized state, multi-nuclear hardware and dedicated transmit/receive coils are necessary, as are specialized, optimized pulse sequences that account for the physical properties and relaxation times of the target nuclei [153]. Unlike conventional ^1^H-MRS(I), heteronuclear approaches are technically demanding, given their lower intrinsic sensitivity, rapid signal decay in hyperpolarized probes, and the need to balance temporal, spatial, and spectral resolutions against SNR constraints. Even though thermally polarized tracers eliminate the need for expensive hyperpolarization equipment, multinuclear acquisition hardware remains expensive and expertise-demanding. Furthermore, acquisition reproducibility can be easily hampered by field strength, B_0_/B_1_ inhomogeneity, coil sensitivity, implemented sequences, vendor-specific hardware differences, and reconstruction approaches, thereby complicating standardization across imaging centers. Mitigating some of these limitations, indirect detection approaches allow investigation of the metabolic fate of ^2^H- and ^13^C-labeled tracers via labeling-induced changes in conventional ^1^H-MRS(I) spectra [91, 92, 154]. Although these approaches reduce hardware needs and improve accessibility, they are susceptible to spectral overlap and quantification uncertainty. Nevertheless, technical advances continue to improve ^1^H-MRS(I) signal sensitivity, SNR, and spectral reliability, including the development of wireless radiofrequency coils and optimized sequences for glutamate/glutamine separation [155, 156]. Similarly, the SNR of ^13^C-MRS(I) can be easily and cost-effectively boosted with the recently developed coil-based methodology to obtain ^13^C sensitivity profiles at the endogenous ^24^Na frequency [157]. However, these approaches introduce additional assumptions into the modeling of compartmentalization and tissue composition, increasing uncertainty in biological interpretation.

For HMI, inherently short signal lifetime can be addressed by newly developed fast-imaging methods that combine EPI with a spatial-spectral radiofrequency pulse [158]. Additional examples include semi-localization via an adiabatic selective refocusing (semi-LASER) sequence and an optimized 3D stack-of-spirals balanced steady-state free precession (bSSFP) sequence for obtaining hyperpolarized ^13^C spectra [159, 160]. The implementation of these sequences improves temporal resolution, sometimes at the expense of spatial resolution and spectral bandwidth. An alternative approach to improving SNR is metabolite-specific acquisition, in which the hyperpolarized probe is acquired at high spatial resolution, while downstream metabolites are acquired at lower spatial resolution but with enhanced SNR [161]. Metabolite-specific acquisition addresses the trade-off between spatial resolution and signal sensitivity, yet it is complicated by low SNR, reconstruction, and quantification uncertainties. Importantly, the clinically used acquisition hardware and pulse sequences fall under medical device regulatory frameworks, such as the European Union Medical Device Regulation (MDR) and the FDA, delaying their immediate use [162, 163]. Therefore, widespread MRS(I) implementation is limited by technical optimization, lack of validation, standardization, and reproducibility assessment, as well as regulatory approval.

### Preprocessing and spectral reconstruction

Following data acquisition, MRS(I) spectra are extensively preprocessed using frequency and phase correction, spectral alignment, baseline correction, eddy-current correction, apodization, and noise reduction to ensure reliable subsequent metabolite quantification. Correctly implemented preprocessing is important, especially in heteronuclear MRS(I), where low SNR, spectral overlap, rapid signal decay, and motion sensitivity increase susceptibility to reconstruction artifacts and quantification inaccuracy. Above all, the choice of preprocessing influences metabolic quantification, modeling outcomes, and subsequent biological interpretation. Consequently, the number of preprocessing choices and the lack of a standardized approach limit reproducibility and comparability across sites, leaving consensus on reconstruction and preprocessing workflows unresolved.

Much research focuses on enhancing spectral quality and reconstruction accuracy, including spatially constrained models for SNR enhancement [164], wavelet-feature-assisted denoising and spectral classification frameworks [165], and a Monte Carlo-validated noise-reduction pipeline for ultra-high-field acquisition [166]. Further advances include new approaches to radiofrequency B_1_ mapping and radiofrequency coil-based distortion correction for ^13^C spectra [167–169]. Metabolic map reconstruction and spatial specificity can be further improved by integrating ^1^H-MRS(I)-derived structural information or by utilizing patch-based algorithms that incorporate compartmentalization information [170, 171]. However, denoising and reconstruction approaches rely on balancing noise suppression, spectral fidelity, and spatial resolution, as excessive denoising or spatial regularization can obscure subtle metabolic differences, introduce oversmoothing, and distort signal dynamics, thereby affecting subsequent metabolite quantification and kinetic modeling. Acquisition-specific choices affect reconstruction performance, which varies across field strengths, scanners, and patient cohorts. Therefore, the lack of standardization, preprocessing, and reconstruction approaches, combined with a vast number of available methodological choices, hampers reproducibility and broad implementation of MRS(I) despite continuous technical advances.

The rise of artificial intelligence (AI), particularly machine- and deep-learning-based reconstruction approaches, has significantly improved MRS(I) workflows by increasing accuracy and speed [172–177]. For instance, deep-learning techniques trained on simulated MRS(I) data can reconstruct high-quality metabolic images from accelerated scans, thereby making ^13^C-HMI acquisition faster and more practical [178]. Similarly, machine-learning-based approaches may partially replace conventional voxel-wise curve fitting by estimating metabolic rate constants, thereby yielding higher-quality metabolic maps [179]. Nevertheless, AI-based techniques have major limitations, including the fact that many models are trained on simulated or highly tailored datasets that fail to capture physiological heterogeneity, hardware-dependent effects, motion artifacts, and pathological heterogeneity in clinical populations. Importantly, AI-based models have not been generalized and validated across hardware vendors, field strength, and acquisition protocols. Furthermore, it cannot be ruled out that the deep-learning-based reconstruction approach introduces hallucinations, oversmooths the data, and hampers biological insights.

For HMI, additional challenges during reconstruction arise from rapid signal decay, sensitivity to acquisition timing, and transient nonequilibrium signals. The accuracy of kinetic modeling from hyperpolarized spectra has been shown to depend on spectral resolution, and some studies have introduced T_2_^*^ corrections to improve the accuracy of metabolic flux estimates [180, 181]. Nevertheless, parameter identifiability, quantification, and reproducibility remain highly dependent on reconstruction assumptions. Collectively, these limitations and considerations highlight the complexity of developing standardized preprocessing pipelines and uncertainty-aware reconstruction frameworks essential for broad dynamic MRS(I) implementation.

### Metabolic modeling

Following preprocessing and quantification, metabolite levels are incorporated into kinetic modeling, typically based on systems of ordinary differential equations, in which endogenous metabolites-, tracer-, or probe-derived signals are fitted to biochemical pathway models. As dynamic MRS(I)-derived spectra are time-resolved, (one-, two-, or multi-)compartmental models enable estimation of metabolic fluxes, turnover rates, and exchange kinetics between compartments (Fig. 1) [90, 182]. By leveraging kinetic modeling, dynamic MRS(I) provides a quantitative opportunity to probe glycolytic activity, oxidative metabolism, metabolic reprogramming, and bottlenecks in NDDs. Yet, accurate modeling depends on reliable probe administration, precise measurements of blood isotopic enrichment, robust metabolite quantification, and appropriate selection of the kinetic model and assumptions.

Dynamic MRS(I)-derived metabolic fluxes are subjected to model-dependent methodological limitations. Parameter identifiability is a challenge, as low SNR or limited temporal resolution can yield multiple combinations of kinetic parameters that fit the observed signal equally well. Additionally, compartmental models simplify the complexity of metabolism by assuming homogeneous tracer distribution, constant compartment exchange rates, and simplified neuronal-glial interactions, ignoring regional cellular heterogeneity. Therefore, derived metabolic changes cannot be attributed to specific biochemical pathways or cellular compartments.

Metabolic modeling is further constrained by confounding factors, such as age, which have been shown to affect lactate production and acetyl-CoA generation even in healthy adults [183]. Physiological differences in tissue perfusion further confound tracer delivery readouts, meaning that changes in tracer-delivery signal may reflect differences in delivery rather than changes in metabolic fluxes. The confounding effects of physiological perfusion can be eliminated by co-injecting metabolically inert probes such as hyperpolarized [1-^13^ C]urea, [1-^13^ C]hydroxymethyl, or [1-^13^ C]butanol, which are not metabolized and provide a signal that reflects tracer delivery and clearance alone [184]. However, such approaches add to the strain and complexity of the measurement. The obtained ^13^C spectra can be normalized using exhaled ^13^CO_2_ measurements, thereby reducing data variability and improving biochemical specificity in a low-cost experimental extension [185], while the metabolic clearance rate model enables simultaneous measurements of metabolism and perfusion [186].

Nevertheless, data interpretation remains limited, as observed metabolic changes cannot be readily attributed to specific cellular processes due to the intrinsic heterogeneity of brain tissue and the differential contributions of various cell types across brain regions. Additionally, validating in vivo metabolic fluxes is challenging, as ground-truth reference measurements are limited by the difficulty of directly verifying compartment-specific metabolism in human brain tissue. However, some of these limitations are being gradually addressed through the development of new tools. For example, available Python-based tools enable the integration of spatially resolved spectra with whole-brain maps of GABA and glutamate receptors, thereby enhancing the biological interpretability of MRS(I)-derived data [187]. Nevertheless, such approaches are only the initial steps towards improving MRS(I)’s utility and interpretability and will require further standardization of modeling frameworks and assumptions, multicenter reproducibility, and biologically validated interpretation.

### Remaining challenges

Dynamic MRS(I) holds great potential to advance our mechanistic understanding of metabolic alterations in NDDs; however, its use is hampered by technical, biological, modeling, and translational barriers.

#### Technical barriers

Existing comparative literature on dynamic MRS(I) is scarce, with few tracer administration protocols investigated (dosages, timeframes, regimes), often in small cohorts (*n* < 10). Consequently, researchers must tailor protocols to experimental settings and research questions, with little standardization of tracer dosage, acquisition timing, reconstruction workflows, and modeling assumptions, limiting reproducibility and comparability across patient populations. From a technical perspective, the use of thermally stable tracers requires prolonged acquisition times (> 1 hour) to achieve sufficient SNR, increasing susceptibility to motion artifacts. Even minor head motion increases susceptibility to frequency drift, spectral broadening, and voxel misregistration, reducing spectral quality and compromising metabolite quantification. This issue is especially prominent in the elderly, pediatric and patients with movement disorders such as PD and MSA, underscoring the need for patient stratification when considering dynamic MRS(I). Consequently, fast-acquisition sequences and motion navigators are being developed and validated, though most solutions are currently applicable only to ^1^H-MRS(I) [188–194]. Additionally, shifting from single-voxel MRS to whole-brain coverage, alongside the development of molecularly targeted MRS(I), may reduce sensitivity to localized motion artifacts [195–197]. While addressing one limitation, these developments introduce additional computational and reconstruction demands, as well as modeling assumptions, that may affect quantitative reproducibility.

#### Biological confounders

Study design should also consider interindividual factors, including metabolic rate, tracer absorption, sex, body weight, systemic metabolism, and cerebral perfusion. If not properly accounted for in metabolic modeling, these variables may alter isotopic enrichment, tracer kinetics, and metabolic fluxes, complicating comparability across subjects. Notably, an incomplete understanding of baseline brain metabolism under physiological conditions poses a major limitation. Without a comprehensive characterization of age-, sex-, and metabolic state-dependent normative metabolism, differentiating disease-associated alterations from physiological variation is exponentially harder. Large-scale, systematic characterization of brain metabolism in healthy volunteers is necessary to establish and validate a normative reference framework to improve the interpretation of disease-associated metabolic reprogramming and bottlenecks.

#### Modeling limitations

As already mentioned, dynamic MRS(I)-derived metabolic insights are fundamentally model-dependent and have their own set of challenges. The heterogeneity of preprocessing choices and spectral fitting strategies remains to be addressed. Parameter identifiability, instability of kinetic parameters, and model overfitting are the main challenges, especially with small sample sizes and data with limited temporal resolution. Dynamic MRS(I)-derived metabolic insights should not be interpreted in isolation but rather integrated with complementary data from PET, ex vivo biochemical assays, genetic profiling, transcriptomics, and cell cultures, thereby improving mechanistic interpretation and validating compartment-specific observations.

#### Translation barriers

Although dynamic MRS(I) is constantly advancing, it relies on expensive multinuclear hardware and hyperpolarization infrastructure, and its use is further limited by the demanding operational burden. Widespread MRS(I) utilization is further slowed by the regulatory approvals of tracers, hyperpolarized probes, acquisition hardware, and pulse sequences. Above all, improvements in signal sensitivity and acquisition speed may be insufficient for broad clinical implementation without simultaneous harmonization of preprocessing pipelines, biological validation of quantification methods, and multicenter harmonization.

## Conclusions

In this review, we described the workflow and spectrum of MRS(I) approaches for in vivo metabolic measurements. At present, dynamic MRS(I) is confined to a small number of institutions, with its use predominantly limited to small patient cohorts with NDDs. Nevertheless, these early insights provide valuable mechanistic understanding, contributing to technical development, biological validation, baseline knowledge, and the gradual harmonization of these techniques, laying the foundation for broader implementation and future clinical utilization. Addressing the limitations presented improves the utility of dynamic MRS(I), which holds great clinical promise in three main domains: disease diagnostics and monitoring, patient stratification, and therapeutic efficacy assessment. Brain function relies heavily on glucose and amino acid metabolism, with some pathological changes either driving disease development or occurring as part of a cascade of downstream pathological events. Its investigation bypasses the need for preclinical disease modeling and replication, with minimal burden on patients, offering a valuable approach for early diagnosis and longitudinal disease monitoring, one already used in oncology research [198, 199]. Dynamic MRS(I) also shows promise for patient stratification, facilitating selection based on patients’ unique metabolic profiles, tailoring the therapeutic approach, maximizing efficacy, and improving quality of life. An additional application is the evaluation of therapeutic efficacy, either directly by investigating on-target metabolic effects in patients or indirectly by assessing beneficial or detrimental metabolic changes.

To summarize, the shift from a static to a dynamic MRS(I) provides a transformative clinical tool for real-time investigation of brain metabolism, with significant potential across various domains of clinical research. Ongoing advances in MRI hardware, the development of cheaper, *in vivo-*friendly probes, and growing expertise in its application are steadily laying the foundation for its integration into everyday practice.

## Data Availability

Not applicable.

## Abbreviations

^18^FDG: ^18^F-fluorodeoxyglucose
^2^H: Deuterium
AD: Alzheimer’s disease
ADP: Adenosine diphosphate
AI: Artificial intelligence
ALS: Amyotrophic lateral sclerosis
a-KG: alpha-ketoglutarate
ATP: Adenosine triphosphate
BBB: Blood-brain barrier
bSSFP: balanced steady-state free precession
CBD: Corticobasal degeneration
COQ8A: coenzyme Q8A
CSF: Cerebrospinal fluid
CRT: Density-weighted concentric ring trajectory
CSI: Chemical shift induction
DL: Deep learning
DMI: Deuterium metabolic imaging
DNP (dDNP): Dynamic nuclear polarization (dissolution DNP)
EPI: Echo planar imaging
FAO: Fatty acid β-oxidation
FDA: Food and Drug Administration
FID: Free induction decay
GABA: 𝛾-aminobutyric acid
Glc: Glucose
Glu2/Glu4: Glutamate labeled at 2nd/4th position
Glx: A combination of glutamate and glutamine
GMP: Good manufacturing practice
GRAPA: Generalized autocalibrating partially parallel acquisition
GRASE: Gradient and spin echo
HD: Huntington’s disease
HMI: Hyperpolarized metabolic imaging
HMQC: Heteronuclear multiple quantum coherence
HP: Hyperpolarized
iP: inorganic phosphate
ISIS: Image selected in vivo spectroscopy
MAO-B: monoamine oxidize B
MDR: Medical device regulation
ML: Machine learning
MRI: Magnetic resonance imaging
MRS: Magnetic resonance spectroscopy
MRSI: Magnetic resonance spectroscopic imaging
MSA: Multiple system atrophy
NAA: N-acetyl-L-aspartate
NAD^+^/NADH: Oxidized/reduced forms of nicotinamide adenine dinucleotide
NDDs: Neurodegenerative diseases
NMR: Nuclear magnetic resonance
NOE: Nuclear Overhauser effect
OXPHOS: Oxidative phosphorylation
PCr: Phosphocreatine
PD: Parkinson’s disease
PET: Positron emission tomography
pH_2_: parahydrogen
PHIP: Parahydrogen-induced polarization
ppm: parts per million
PSP: Progressive supranuclear palsy
SABRE: Signal amplification by reversible exchange
SAR: Specific absorption rate
SENSE: Sensitivity encoding
Semi-LASER: Semi-localized by adiabatic selective refocusing
SNR: Signal-to-noise ratio
T: Tesla
TCA: Tricarboxylic acid
VOI: Volume of interest
